# Electrospun Nanofibrous Scaffolds: Review of Current Progress in the Properties and Manufacturing Process, and Possible Applications for COVID-19

**DOI:** 10.3390/polym13060916

**Published:** 2021-03-16

**Authors:** Mohamed Kchaou, Mohammed Alquraish, Khaled Abuhasel, Ahmad Abdullah, Ashraf A. Ali

**Affiliations:** 1 Department of Mechanical Engineering, College of Engineering, University of Bisha, P.O. Box 001, Bisha 67714, Saudi Arabia; malqraish@ub.edu.sa (M.A.); kabuhasel@ub.edu.sa (K.A.); ahassanein@ub.edu.sa (A.A.A.); 2 Department of Civil Engineering, College of Engineering, University of Bisha, P.O. Box 001, Bisha 67714, Saudi Arabia; ahmed.abdalla@aswu.edu.eg; 3 Department of Civil Engineering, Faculty of Engineering, Aswan University, Aswan 81542, Egypt

**Keywords:** electrospinning technique, nanofiber scaffolds, bio-solution, innovative process, new biotechnology applications, COVID-19

## Abstract

Over the last twenty years, researchers have focused on the potential applications of electrospinning, especially its scalability and versatility. Specifically, electrospun nanofiber scaffolds are considered an emergent technology and a promising approach that can be applied to biosensing, drug delivery, soft and hard tissue repair and regeneration, and wound healing. Several parameters control the functional scaffolds, such as fiber geometrical characteristics and alignment, architecture, etc. As it is based on nanotechnology, the concept of this approach has shown a strong evolution in terms of the forms of the materials used (aerogels, microspheres, etc.), the incorporated microorganisms used to treat diseases (cells, proteins, nuclei acids, etc.), and the manufacturing process in relation to the control of adhesion, proliferation, and differentiation of the mimetic nanofibers. However, several difficulties are still considered as huge challenges for scientists to overcome in relation to scaffolds design and properties (hydrophilicity, biodegradability, and biocompatibility) but also in relation to transferring biological nanofibers products into practical industrial use by way of a highly efficient bio-solution. In this article, the authors review current progress in the materials and processes used by the electrospinning technique to develop novel fibrous scaffolds with suitable design and that more closely mimic structure. A specific interest will be given to the use of this approach as an emergent technology for the treatment of bacteria and viruses such as COVID-19.

## 1. Overview of the Electrospinning Technique

### 1.1. State of the Art

Electrospinning is widely attractive to industry and researchers for its scalability, versatility, and potential applications in many fields [1,2]. It is considered one of the most suitable techniques for fabricating nanofibrous scaffolds, which are known for their high physical porosity and huge potential to mimic defects, such as bone defects [3,4,5]. In terms of geometry, the diameter of each electrospun fiber depends on the polymer specificity and electrospinning processing parameters [3]. The electrospinning set-up consists of a high voltage source, an infusion syringe pump, and a collector; the collector might be stationary or portable metal or a coagulating bath [4,5,6]. Electrospinning technique produces more thinner, smoother, and folded scaffolds and achieves more uniform drug distribution with less residual liquid than the solvent casting [7]. Therefore, electrospinning induces well-controlled drug release profiles. In the next paragraph, the potential applications of nanofiber scaffolds will be further highlighted.

Nowadays electrospinning is a well-known technology and it has been under a thorough investigations. One of the earliest studies of the electrified jetting phenomenon has been published by Zeleny [8]; in this paper the role of the surface instability in electrical discharges from charged droplets has been studied. A series of patents from 1934 to 1944 has been published by Formhals [9,10,11,12]. These patents are mainly describing an experimental setup utilizing electrostatic forces to produce fine dried polymer filaments. Another apparatus for the production of patterned, ultrathin, low weight, non-woven fabrics by using electrical spinning has been filled by Simons [13] in 1966. A deformation of a charged liquid meniscus has been studied by Taylor [14,15,16,17], Taylor described a conical stable geometry at the end of the meniscus which is now known as a Taylor cone. In 1971, Baumgarten [18] used electrospinning apparatus to produce ultra-fine acrylic fibers with diameters below 1.1 μm down to 500 nm. Although the electrospinning process has been extensively studied by many researchers since then for several decades in the literature, many parameters are still under investigation and not yet completely understood [19].

A research team in Akron University, USA lead by Professor Reneker [20] reintroduced the electrospinning technique to make submicron fibers from different types of synthetic and natural polymers. Yaril’s team reported the production of hollow nanotubes by co-electrospining two polymer solutions (PCL and PMMA nanofibers) [21,22]. Researchers particularly highlighted the desorption limited mechanism of release from polymer nanofibers [23] and discussed electrospinning jets in the form of a polymer fiber with a diameter that can often be conveniently stated in nanometers [24].

Polyacrylonitrile (PAN) and copolymers of PAN are considered a very important candidate for electrospinning as they have commercial/ technological applications. Among various precursors of carbon nanofibers (CNFs), PAN is considered the best candidate, mainly due to its high carbon yield (up to 56%), flexibility and the ease of its heat treatment stabilization stage to form a stable Zigzag structure during nitrile polymerization [25,26,27,28,29,30,31,32,33]. Also, PAN has excellent characteristics, such as spinnability, environmentally friendly nature and varieties of commercial applications.

Inagaki et al. [34] describes the chemistry and applications of CNFs. Barhate and Ramakrishna [35] published a review on nanofibers as a filtering media for tiny materials. Li and Xia [36] discussed about the trends in nanofibers with emphasis on electrospinning techniques to produce nanofibers. PAN nanofibers and carbon nanotube (CNT) reinforced PAN nanofibers were successfully electrospun [37]. In our research team, Ali and et al. [38,39,40,41,42,43,44,45,46,47,48,49] published a series of publications studying the characteristics of the electrospun PAN/N, N-dimethylformamide (DMF) polymer solution using different types of collectors. Hot pressing technique has introduced as well to electrospun PAN nanofibers with and without nano reinforcements to produce carbon nano fibers. In their work optimization of the process for PAN nanofibers has been introduced by response surface methodology (RSM). 

RSM has been used as a successful tool for optimizing the process in both polymer electrospinning and polymer hydrogels [50,51,52]. Process optimization of nanofibers has been investigated by RSM in order to predict the electrospinning parameters affecting the producing nanofiber diameter in order to achieve minimum fiber diameter in nano level. A quantitative relationship between electrospinning parameters and the responses (mean diameter and standard deviation) was established and then the final multi-layers structure of nanofibers and nanoparticles has been achieved for a controlled and robust process [53,54,55]. 

In the last decade, nanofibers have been generated by electrospinning under pressurized CO_2_. In fact, an evolution of the traditional technique was clarified by several researchers by adding CO_2_ in the liquid polymeric solution [56,57,58]. The presence of CO_2_ in the solution reduced the surface tension of the liquid to be processed and its viscosity. Wahyudiono et al. [59] demonstrated that CO_2_ dissolved in the starting liquid polymeric solution introduced greater flexibility in the process with respect to the classical technique by the production of micro- and nanofibers of poly(vinylpyrrolidone (PVP) through the adoption of a more advanced configuration of the process. Baldino et al. [60] reported a more developed technique called supercritical assisted electrospraying (SA-ESPR), in which the addition of supercritical CO_2_ (sc-CO_2_) to a starting polymeric liquid solution produced a controlled size of micro- or nanoparticles. Attempts to produce nano-composite (polymer + drug) using this electrohydrodynamic process have shown encouraging results in the last years [61].

### 1.2. Electrospinning Parameters

Electrospinning parameters are essential to understand not only the nature of electrospinning but also the conversion of polymer solutions into nanofibers through Electrospinning. These parameters that affecting electrospinning process and electrospun fiber diameters can be classified under three main categories named as: polymer solution, processing, and environmental.

These parameters can affect morphological characteristic of electrospun fibers as well as its size. A summary discussion of these parameters and their effect on fiber characteristics will be presented:

#### 1.2.1. Solution Parameters

##### Concentration and/or Berry’s Number

The concentration or Berry’s number of a polymer solution or melt play a crucial role in fiber formation during the electrospinning process. Four critical concentrations from low to high should be noted:

1. When the concentration is very low, micro to nano beads will be obtained. Here, electrospraying occurs instead of electrospinning, owing to the low viscosity and high surface tensions of the solution [62].
2. As the concentration increases, a mixture of fine beads and microfibers will be obtained [63,64,65].
3. At a specific Berry’s number and when the concentration is adequate, smooth nanofibers can be obtained.
4. At high concentrations, no nanofibers are obtained; helix-shaped, coil structures, and microribbons will be observed [66].

In general, as the concentration or Berry’s number of solution or melt respectively increases the fiber diameter increases within the spin-able range. 

##### Molecular Weight

Molecular weight of the polymer reflects the degree of polymer chain entanglement in solutions accordingly indicating solution viscosity. In case of keeping the concentration fixed, as molecular weight of the polymer decreases beads formation rather than smooth fiber is the probability. Enhancing molecular weight value results in smooth fiber. Increasing in molecular weight results in micro-ribbon formation [67].

It is also important to note that by increasing molecular weight even with low concentration micro-ribbon could be formed [68,69].

##### Intrinsic Viscosity

Viscosity of polymer solution is considered one of the most important parameters affecting electrospun fiber diameter and morphology. There is a suitable viscosity for the electrospinning to form fiber [70,71]. Group of publications on the correlation between polymer solution viscosity and formation of electrospun fiber have been published [67,72,73]. Concentration, viscosity and polymer molecular weight all are correlated to each other and one parameter has been used to describe them all named Berry’s number it measures the degree of chain entanglement inside the solvent and can be calculated by the product of concentration by intrinsic viscosity. Ali and et al. [38,39,40,41,42,43,44,45,46,47,48] correlates such parameter with the optimization of electrospun PAN fibers.

##### Surface Tension

Surface tension is important factor in electrospinning. In 2004, Yang and Wang investigated the influence of surface tensions on electrospun PVP fibers with three different types of solvent namely ethanol, DMF, and MC [66].

As the surface tension of the solution decreases, beaded fibers can be converted into fine fibers [74,75,76,77,78,79,80]. Also, in this study, the surface tension and solution Viscosity can been adjusted by changing the mass ratio of solvents mix and fiber morphologies.

If all other conditions are constant, surface tension identifies the upper and lower boundaries of the electrospinning window [77,79].

##### Conductivity/Surface Charge Density

Solution conductivity is specified by polymer bond type, solvent solubility parameter and any other additives such as salt due to its ionic bond type. Usually, natural polymers are polyelectrolytic in nature, in which the ions increase the charge carrying ability of the polymer jet, subjecting to higher tension under the electric field, resulting in the poor fiber formation on the other hand the synthetic polymers are tending to form good fibers [80]. With the aid of ionic salts, nanofibers with small diameter can be obtained [81]. 

Sometimes high solution conductivity can be also achieved by using organic acid instead of regular solvent. Hou et al [81] used formic acid as the solvent to dissolve nylon 6,6 and obtained nano fibers of 3 nm diameter with beads. In their study, small amount of pyridine has been added into the solution to enhance capacity of solution to carry charge aiming to eliminate the beads by increasing the conductivity of the solution. In general, increase in the solution conductivity the more possibility of formation of thinner fibers especially in synthetic polymers.

#### 1.2.2. Processing Parameters

##### Voltage

One of the important parameter in the electrospinning process is the applied voltage value. Only if the applied voltage overcomes the surface tension of the polymer meniscus then an ejected jet comes out from Taylor Cone. However, increasing in the value of applied voltage does not in the favor of the characteristics of the electrospun fiber diameter or/and morphology. Reneker and Chun [82] have proved that there is not much effect of electric field on the diameter of electrospun polyethylene oxide (PEO) nanofibers. Several groups suggested that higher voltages than required to form ejected charged jet implies to form large fiber diameter. Zhang et al [79] theoretically studied the effect of voltage on morphologies and fiber diameters distribution with poly (vinyl alcohol) (PVA)/water solution in a theoretical modeling approach.

Another research groups showed that higher applied voltages may be increase the electrostatic repulsive force on the charged jet and accordingly smaller fiber diameter can be formed. Yuan et al [83] investigated the effect of applied voltage on morphologies and fiber alignment with polysulfone (PSF)/DMAC/acetone as model. In addition to those phenomena, some groups also demonstrated that higher voltage offers the greater probability of beads formation [62,84,85].

Definitely and at the end of the previous discussion voltage does influence fiber diameters. However, the significance level differs according to polymer type, solution concentration and finally the distance between the tip of the spinneret and the nearest point on the collector [86].

##### Flow Rate

Another important processing parameter is the polymer solution flow rate. Generally, lower flow rate is more recommended as the polymer solution will get enough time for polarization. If the flow rate is very high, bead fibers with thick diameter will form rather than the fine fiber. That can be easily explained due to the short drying time period that the travelling jet has taken until it reaches the collector. Yuan et al [83] studied the effect of the flow rate on the morphologies of the PSF fibers from 20 % PSF/DMAC solution at 10 kV. In their study, bead fibers with thicker diameters obtained as the flow rate is 0.66 mL/h.

##### Collectors

Metal collector is acting as the conductive substrate (ground) to collect the charged fibers. Aluminum foil is used as a cover sheet attached to the stationary metal grounded screen but it is difficult to transfer the collected nanofibers to other substrates for various applications. With the need of fibers transferring, diverse collectors have been developed including cone [87], pin [88], metal grids [89], parallel or gridded bar [46], rotating rods, cylinder or wheel [90], wet coagulating bath [91].

##### Distance or Spinning Height (H) between the Collector and the Tip of the Spinneret

Traveling distance between the tip of the charged spinneret and the nearest point of the grounded collector certainly affect the fiber diameter and its morphologies [73]. Conceptually, as the travelling bath increases more time is consumed by the travelling jet and more chance and possibility of getting rid of the solvent and consequently drying of the jet into fine fiber on the collector surface is expected [83].

#### 1.2.3. Environmental Parameters

Temperature and Humidity are two important environmental parameters that can also affect the fiber diameters and its morphologies. Mituppatham et al. [92] proved that as temperature increases smaller and thinner fiber diameter is collected.

Low humidity tends to dry the solvent totally and increase the velocity of the solvent evaporation. On the contrary, high humidity will lead to thicker fiber diameters. Casper et al. [93] demonstrated that the variety of humidity can also affect the surface morphologies of electrospun polystyrene (PS) fibers.

## 2. Potential Applications of Electrospun Nanofiber Scaffolds (ENS)

### 2.1. Tissue Engineering Applications

Tissue engineering integrates science of biology and medicine to design artificial organs for regeneration of tissue function [94,95]. In order to mimic the extracellular matrix and provide tissue with oxygen and nutrient circulation, functional tissues were fabricated from several materials and specific structures, particularly nanofiber scaffolds [96,97]. In fact, nanofiber scaffolds are widely used in tissue repair, whether soft or hard regeneration [98]. Faced with therapeutic problems, damaged ligament, fracted bone cartilage, and blood vessels were restored, taking advantage of ENS to repair orthopedic tissues and/or develop organs with high similarity in term of characteristics, properties, and design [99,100,101]. Exploring their characteristics, ENS is used for wound healing. In fact, it is considered a solution for the loss of skin integrity caused by injury or illness. It provides additional biological stimuli to support cell and tissue function. As consequence, substantial psychological balance is noticed in the tissue-engineered skin patients [102,103,104].

### 2.2. Drug or Protein Delivery

Exploring the ability to control biomaterial properties (geometry, fiber alignment fiber diameter, composition, etc.), ENS are used to incorporate drugs/proteins into scaffold [104]. In fact, known for its large surface-to-volume ratio, it was demonstrated by several researchers that ENS could be a perfect vehicle for drug delivery, either by dissolving the drug into the electrospinning solution or by mixing the drug with this solution when the drug is not soluble [105,106,107]. As consequence, the drug contained inside in the fiber will be released. Several solutions are used to master the adsorption of a drug, particularly when the solubility of the drug is limited. Pillay et al. [108] proposed an immersion step of nanofibers into a drug solution to let the drug molecules chemically and physically bond or attach to the ENS. Yoo et al. [109] demonstrated that nanofiber can biomimetically bind to a drug after electrospinning by controlling physical adsorption (both when we use simple physical adsorption or nanoparticle assembly on the surface or even layer by layer-by-layer multilayer assembly). Chemical adsorption methods can be explored by surface activation (such as plasma or ultraviolet treatments) or bioactive molecule immobilization [110,111].

The reliability of the drug delivery application is conditioned by the drug–polymer compatibility, as well as the foreign body’s interaction with natural organ or tissue [112,113]. Varied results were noticed using drugs with different bioactivities, such as antimicrobial [114,115], anticancer [116,117], anti-inflammatory [118,119], cardiovascular [120,121], or antihistamine [122,123] drugs. Another factor that can influence the drug-release kinetic is the type of the polymer used as a matrix for the nanofiber’s elaboration and its architecture (sandwiched with microparticle, sandwiched with microfibers, etc.) [124].

For protein delivery, surface immobilization of bioactive molecules has been used to load proteins into ENS fibers to protect the molecules from the effects of high voltage [125]. The method consists of the fixation of the protein scaffolds surface using suitable chemical conjugation with corresponding functional groups, such as carboxylate [126]. It was reported by Tigli et al. [127] that cell fate powered by peptides is conditioned by the control of the drug feeding ratio.

Cancer has become a leading disease, causing human death worldwide [128]. Cancer is responsible for 15% of human deaths worldwide, with 1.5 million new cases expected annually. Chemotherapy, hormonal therapy, radiation therapy, immunotherapy, and surgery are the current standard methods of treatment. Chemotherapeutic drugs in clinical use such as 5-fluorouracil (5-FU), cisplatin, carboplatin, paclitaxel, gemcitabine (Gemzar), etc. are characterized by either poor bioavailability, poor selectivity and specificity, or by liver accumulation and fast renal clearance [129]. Nanotechnology’s evolution offers promise to tackle these problems. For instance, anticancer drug-loaded polymeric nanoparticles (NPs) are advantages to traditional anticancer drug formulations in terms of diminishing the adverse effects of drugs and enhancing their therapeutic efficacy. Generally, through the enhanced permeability and retention effect, some NPs are accumulated preferentially in tumors. Additionally, electrospun nanofibers (NFs) formulation became a promising techniquein the delivery of drug [130] and wound dressing [131]. Due to their high porosity and important surface area to molar ratio that mimics the extracellular matrix, they gained their importance in biomedical engineering [132]. Examples of these formulations include encapsulation of anticancer drugs into biodegradable polymeric nanofibers; 5-FU and salinomycin on poly(lactic-co-glycolic acid) (PLGA) [133]; Paclitaxel on PCL [134]; Doxorubicine HCL-block-poly(ethylene glycol)-block-poly(L-lactide) (HCl on PEG–PLLA) [135], hydroxycamptothecinon (Poly(lactic acid)−Poly(ethylene oxide)) PLA–PEG [136].

Pawłowska et al. [137] highlighted the development of smart drug delivery systems using the stimuli-responsive electrospun nanofibers. Their developed nanoctruture pillows have the specificity of fast photothermal responsiveness for near-infrared (NIR) light-controlled on-demand drug delivery. The innovative platform consists of electrospun PLLA loaded with a rhodamine B drug model that encapsulates platonic hydrogel P(NIPAAm-co-NIPMAAm)/AuNR [138]. The researchers demonstrated that this emergent nanotechnology is considered an excellent candidate for achieving on-demand drug release in synergy with photothermal treatment.

### 2.3. Agriculture, Food Industry, and Environment

As reported in many publications, technical reports, and communications, ENS are used in the biosensing [137,138], agriculture protection [139,140], the fermentation food industry [141,142], and biocatalytic remediation of the environment and energy [143,144]. In fact, to monitor food and agriculture using simpler, faster, and less expensive sensitive detection methods of foodborne illness, researchers and developers have exploited the potential given by the electrospinning technique to innovate 1D micro- and nano-biosensors [145]. Taking into account the surface properties, the high level of porosity, and the capability to interact with green elements, nanofibers have been functionalized with several types of nanomaterials such as graphene and carbone nanotubes to acquire the singularity of multifunctional hybrid electrospun nanofibers. These specificities enhance the reactivity of the materials, improve adsorption, and increase the sites’ numbers of catalysts loading and interacting. Zhang et al. [146] proved that the quality of such bio-chemical sensors is attributed to the strong stretching forces associated with electrospinning that induce polymer orientation along the longer axis of the fiber chains. Therefore, high charge-carrier mobility or polarized photoluminescence can be created [147]. Recent laboratories have incorporated new nanomaterials to create what is called ESN-based chemical and hybrid biosensors, which have been applied in the agri-food sector to address food quality and safety [148,149,150]. The conjugated use of polymer, ceramic, and inorganic other materials are reviewed by Mercante et al. [151]. In another branch of this very fertile sector, researchers used biocompatible ESN to entrap bioactive food ingredients, both hydrophilic and hydrophobic types [152]. Besides, they synthesized a food encapsulation method based on ESN to safeguard food from oxidation and damage [153,154]. Other researchers have produced ultra-fine poly(acrylamide) PAM fibers for use as a water super-absorber and soil erosion resistant agent in irrigation systems [155]. By electrospinning a 290 nm fiber diameter, the optimization of Berry’s number, spinning height, and spinning angle on PAM fiber was investigated based on empirical and experimental approaches.

Th application of ESN in environment topics and issues is varied, from energy harvesting/conversion/storage to filtration membranes and catalytic supports [156,157,158]. In fact, specific devices made in ENS were developed and incorporated as solar cells, rechargeable batteries, and fuel cells [159]. Exploring the possibility to electrospin metal oxides and/or carbon nanofibers, they can be used as electrode materials thanks to their properties such as high surface-to-volume ratio, short diffusion distance, and large specific surface area [160]. As a consequence, they are able to rapidly transfer electrons and ions, with a large electrode/electrolyte contact area [161].

As reported by [162,163,164], the mass transport of reactant is feasible using a nonwoven mat of nanofibers. In fact, thanks to their high porosity level and their good interconnection, extensive contact between reactants and active sites on electrocatalysis are provided to fabric fuel cells, particularly from polymer, ceramic, and metal electrospun nanofibers. Their durability and efficiency were confirmed by several researchers and industries [165,166].

## 3. Innovation in Biomimetic Design, Materials Properties, and Structure Architecture

Nowadays, great efforts are focused on innovation in the design of biomimetic electrospun scaffolds for biotechnological applications. Natural and synthetic polymers as well as ceramic and metallic materials have been tested to improve properties and structure architecture.

### 3.1. Biomimetic Design

Biomimicry is a technologically oriented approach focused on creating innovative solutions that are inspired by nature’s wealth [167,168]. In relation to our topic, researchers have concentrated on a few key sources of inspiration, in terms of shape, function, materials, or ecosystem [169,170]. They benefit from the easily tunable compositions and structures of electrospun fibers to successfully biomimic via electrospinning.

Wei et al. [171] reported the design of nacre-inspired porous scaffolds for bone repair. Their work was the result of product design strategy that included electrospinning, phase separation, and 3D printing to elaborate layer-by-layer a composite film with nacre-like structure from nano-platelets and polyamide. Nacre has also inspired newly developed coatings and implants (from simple to complex geometries) that functionalize the biomaterials surface in order to induce desirable biological responses [172,173,174].

Wang et al. developed engineered biomimetic superhydrophobic surfaces of electrospun nanomaterials inspired by the lotus leaf [175]. The same property was explored by investigated silver ragwort leaf and hillock bush leaf [101,176]. Other researchers have focused on the biomimetic of structure and functions of honeycombs, polar bear fur, and spider webs to inspire tissue structure and organ architecture (membrane, bone marrow, etc.) [177,178].

### 3.2. Materials Properties

To electrospin submicrometric fibers, a panel of materials can be used, from a natural source such as gelatin or collagen to a material from a synthetic category such as poly-caprolactone (PCL) and polylactide (PLA), as well as to hybrid types (e.g., PCL blended with collagen) [179,180,181,182,183].

Cell adhesion, migration, spreading, and differentiation are the essential characteristics related to the nanofiber surface’s stiffness. In fact, after implementation, a successful integrated scaffold should provide good mechanical properties in terms of rigidity and flexibility, with an optimal porosity size and calibration [184]. Pennel et al. [185] made a direct relation between the infiltration and vascularization ability of the new material and its continual stability. Liu et al. [186] demonstrated that polylactide co-trim-ethylene carbonate nanofibers showed enhanced efficiencies as scaffold materials for tissue regeneration. Bao et al. [187] developed multifunctional fibrous scaffolds with shape memory specification. They were especially useful for tissue repair. Many other polymers such as chitosan, alginate, and silk fibroin have been explored for the production of healing mechanisms due to their biodegradability, drug release ability, acceptable hydrophilicity, and non-toxicity [188,189,190,191,192,193]. From the animal resource, a polymer solution is developed. Membranes with or without active agents are obtained by electrospining. The cultivation and separation process are operated to create new cells with antibacterial properties.

### 3.3. Architecture

An appropriate cellular environment is required to assume available scaffold architecture. This architecture is the result of various special arrangements (aligned, random, or cross aligned) [194]. These arrangements should ensure neo-tissue elaboration and proliferation, vascularization, and integration without risk to the original tissue [195]. Lutzweiler et al. [196] considered that the most sensitive property of a scaffold’s structure is its ability to diffuse nutrients and metabolites thanks to an optimal pore size and stable architecture. It needs a suitable design for cell migration into scaffold and necessary ligand density on the scaffold surface [197]. According to the principle of regeneration, it should be gradually replaced with extracellular matrix, taking into account the biodegradability of the developed tissue, without any risk of toxication or surrounding organ disturbance [198,199].

Other progress related to the architectural point of view consists of the development of gradient structures to tailor cell orientation and their extracellular matrix deposition [200]. The evolution is related to the fabrication of scaffolding with random and aligned fibers on one section. As a consequence, a graded mechanical property throughout the tissue constructs is possible [201].

## 4. Current Progress on Elaboration Process, Implementation, and Manufacturing

The electrospun nanofibers’ properties such as morphology and diameter are influenced by intrinsic (solution properties) and extrinsic (process and environment) factors [202,203,204]. Dorati et al. [205] reported that solvent concentration, viscosity, electrical conductivity, and elasticity are the most influential parameters on the geometry and morphology of electrospun nanofibers. In fact, to run electrospinning components, a researcher needs a small amount of solution with a suitable concentration to achieve a smooth and uniform nanofiber. Both low or high concentrations could induce a morphological problem or diameter variations according to the interaction of the solvent concentration with the viscosity and the surface tensions effects [206,207]. As consequence, non-uniform shape and non-mastered diameters of nanofibers have a great probability of being detected. Datta and Dhara [208] combined microfabrication and rolling process to design 3D bone grafts based on 2D ENS of synthetic polymer. This approach needed a graphical design of macro-pore that could be created by a laser-engraving machine on EN sheets to facilitate cellular infiltration into the 3D scaffold. Finally, multi-scalar porosity was rolled up to obtain a 3D scaffold, which was associated to the microfabricated nanofiber sheets for a final bio-engineered organ.

As more sophisticated method, a robocasting 3D printing technique was used to develop an electrospun organ with a complex structure, paving a new way to developing unprecedented scaffold microstructure [209,210]. A considerable amount of software is needed to control geometry, architectural structure, and manufacturing parameters to result in more closely mimicking material and highly efficient biomedical scaffolds [211].

Table 1. summarizes the most important applications of the electrospun nanofibrous scaffolds.

## 5. Electrospun Nanofiber Applications for Medical Care in the Coronavirus COVID-19 Pandemic Crisis

COVID-19 is a disease caused by a new kind of coronavirus called severe acute respiratory syndrome coronavirus 2 (SARS-CoV-2) manifested in Wuhan, Hubei, China at the end of 2019 [227]. This novel coronavirus can cause fever, respiratory failure, septic shock, and even death. Researchers believe that SARS-cov2 has zoonotic origin as bat coronaviruses, pangolin coronaviruses, and previously discovered SARS-CoV [228]. To date, COVID-19 is expected to greatly impact society and the economy and to widely change our daily lifestyle [229]. Despite the number of research studies, the high kinetics of publication, and global strategies in relation to this pandemic [230,231,232], development of protection methods and solutions and the search for an efficient, well-controlled, and accurate means of drug dose delivery, especially with regard to the respiratory system of newly infected COVID-19 patients by using biodegradable electrospun polymer, are still considered potential issues until an effective vaccine is developed and made widely available [233].

As soon as the pandemic appeared, Tebyetekerwa et al. [234] innovated unique electrospun nanofibers for filtration membranes in face masks. They proposed the use of durable and yet reliable electrospun nonwoven filters with 10 nm fiber diameter, as the filtration efficiency of polymeric nanofibers mats is excellent and can adsorb submicronic and nanosized organisms [235].

In this context, Zussman’s research group developed a sticker to upgrade surgical masks called “Maya” [236]. This innovative product showed successful results in the protection of respirators, trapping nanometric particles and, thanks to new functionalities given by the electropunk nanofibers, efficiently neutralizing the virus from droplets that might reach the mask surface.

Bin Ding’s team developed other types of masks using nanofibrous membrane-based desiccants for energy efficient humidity control and atmospheric water harvesting [237]. The new technology of a wood-inspired moisture pump was adapted based on electrospun nanofibrous membrane for solar-driven continuous indoor dehumidification.

Other smart masks were developed by DooKim’s group, which was exploring electrospun nanostructure performance [238]. The researchers designed a membrane to use as an additional filter for tissue-based face masks. Thanks to its nanostructured arrangement with 100–500 nm dimeter fibers, this electrospun membrane showed an excellent filtering efficiency even after being hand washed more than 20 times.

Another new generation of masks using electrospun nanostructure was proposed by Sio et al. [236]. They developed smart self-disinfecting face masks based on a multilayer electrospun membrane. They introduced nanoclusters and plasmonic nanoparticles with a hierarchical arrangement to realize chemically driven and on-demand anti-pathogen activities.

Suitable disinfection methods and protocols should be produced so filters can be reused without compromising filtration efficiency. Khanzada et al. [239] developed aloe vera and polyvinyl alcohol electrospun nanofibers for protective clothes. The efficiency of the innovative product was confirmed by using antimicrobial activity tests to check against Gram-positive and Gram-negative bacteria. An optimum composition for high-antimicrobial activity against *S. aureus*, compared with *E. coli* bacteria was patented. For possible use as a fast absorbent carrier of anti-COVID-19 drug delivery, some researchers are trying to prepare scaffolding that would be able to carry such drugs while controlling the degree of a drug’s absorption by the human body—something that would be indispensable [240,241]. However, this new applied nanotechnology is not yet mastered for use with newly discovered drugs.

## 6. Challenge of the Electrospun Nanofiber Scaffolds

Based on our review study, it is clear that electrospun nanofiber scaffolds are still facing many challenges with relation to the choice of materials (properties, performance, etc.), production policy (complexity, process, rate, etc.), and manipulation (scaffold storage, cost, etc.). In some applications, such as drug delivery, the improvement of drug–polymer compatibility is a primary focus for biologists, with the need to ameliorate the rate of matrix hydration and drug diffusion of the fiber-comprising polymer. Besides, ENS encounter some other practical limitations, such as scare cell infiltration and inadequate mechanical strength for load-bearing application, for example. Therefore, researchers are still focused on innovation in design, material, and architecture, as well as innovation in the manufacturing process.

## Figures and Tables

**Table 1:** Most important applications of the electrospun nanofibrous scaffolds in the last decade.

Application	Polymer and Solvent Used	Product Characteristics	Ref.
Cosmetic mask	A siliceous sponge spicules (SSS) and polylactic acid (PLA).	A nanofiber composite (PLA/SSS) of 50–450-nm with enhanced thermal and mechanical properties; a slight enhancement in human foreskin fibroblast cell proliferation; a decent cytocompatibility; and antibacterial	[212]
	A gelatin solution prepared in ethanol extracted from Crude Carissa Carandas fruits (CCE) and incorporating acetic acid.	A smooth and continuous gelatin fibers mats (GFM) with an average diameter of 235.69 ± 10.45 nm. could be obtained with the optimal conditions of 30% (*w*/*v*) gelatin solution, 25% (*v*/*v*) ethanol solution, 30% (*v*/*v*) acetic acid, a fixed electrostatic field strength of 20 kV and a 15 cm distance between spinneret tip and collector. When 15% (*w*/*w*) CCE is used, the CCE-GFM shows high DPPH radical scavenging and tyrosinase inhibitory activity.	[213]
	Anionic surfactants added to a natural biopolymer of galacturonic acid (PGuA) to enable its electrospinning to nanofibers.	Small spindled fibers of 2 to 10 μm length and 287 to 997 nm diameter. Large continuous fibers could be produced when an amount of 10 to 30% of high molecular weight PVA is used.	[214]
Drug Delivery	A poly(vinylpyrrolidone)/PVP electrospun to encapsulate β-carotene dissolved in ethanol.	PVP/β-carotene composite nanofibers of 176 to 306 nm average diameter were able to protect the β-carotene properties	[215]
	A blend of poly (ε-caprolactone) and poly (ethylene oxide) (PCL/PEO) incorporating a nanosized hydroxyapatite (n-HA) to carry curcumin.	A nanofiber material with slow release rate of curcumin and with a high cytotoxicity against breast cancer cell line	[216]
	Chitosan/pullulan carried by a shell of polylactic acid (PLA)	A nanofiber with improved thermal properties and rapid dissolving capability in water.	[217]
Tissue engineering	A platelet-derived growth factor (PDGF-BB) contained within a shell of polylactic acid (PLA) and encapsulated within nanofibers	A 3D scaffolding nanofibers with microporous structure, acceptable mechanical properties and high cell compatibility.	[218]
	Crystalline cellulose (NCC) in a matrix of cellulose acetate (CA) polymer.	a bio-tissues of nanofibers with uniform diameter, moderate thermal properties and improved mechanical properties of 30 MPa tensile strength and 1.597 MPa module of elasticity.	[219]
	A blend of poly(ε-caprolactone), poly(ethylene glycol), poly(ε-caprolactone) (PCEC) along with polylactide (PLA).	A biodegradable polylactide (PLA)/PCEC fibrous membranes compatible with bone tissues.	[220]
Cancer therapy	A poly(ε-caprolactone) (PCL)	A scaffolding system of long nanofibers to carry breast cancer therapy	[221,222]
Wound dressing	A zein/Graphene oxide (GO) blend. The GO is loaded by Tetracycline hydrochloride (TCH).	A nanofiber composite with enhanced mechanical properties and improved release profile.	[223]
Gene delivery	A biopolymer incorporating nano-hydroxyapatite (nHAp) modified with linear polyethylenimine (LPEI), and poly(ε-caprolactone) (PCL).	A homogeneous and cohesive composite with structural characteristics, swelling and degradation behavior dependent on the size and amount of the included inorganic particles.	[224]
Filter media.	A poly(ε-caprolactone) (PCL)	Nanofibers (NF) of average diameters of 180 and 234 nm with improved bioprotective activity and filtration efficiency	[225,226]

## Data Availability

Data sharing not applicable.
